# Dysentery—Lilium Tigrinum

**Published:** 1883-12

**Authors:** G. M. Pease

**Affiliations:** San Francisco


### CLINICAL BUREAU.

### DYSENTERY.—LILIUM TIGRINUM.

G. M. PEASE, M. D., SAN FRANCISCO.

July 8th called to see Mrs. F., suffering with dysentery. Stools were very frequent and of a bloody mucus; almost constant urging and much backache. When a stool has been had, there remains a feeling as if more would pass. Wakens about 3 A. M. and cannot go to sleep again for several hours, and then sleeps very soundly. This symptom is an old one. Nux. vom.$^{200}$ was given; but, upon a visit made in the evening, she was found to be worse, rather than better, there being a colic added to the symptoms of the morning. The mouth was dry and a constant thirst for large quantities was present. She longed to keep quiet, as the slightest movement produced an aggravation which made her particularly ill-natured.

Bryonia$^{200}$ was administered with the expectation that relief would certainly follow; but upon my next visit I found no improvement, the passages being more bloody and occurring about every thirty minutes. Mercurius was then given, but followed by no good results.

Carefully reviewing the case, I found a mental symptom which led my thoughts toward another remedy. She had a restless, hurried feeling, as if she must attend to some very important duties, but which she was conscious of being unable to do. There was a tenderness over the region of the left ovary, with a downward pressure, as if everything would come out of the vulva, and a feeling as if she must hold herself up in that region whenever she went to stool, also frequent desire to urinate.

Although Lilium tigrinum was the remedy that came into my mind, it lacked the bloody stool and many of the other symptoms; but as she had the mental, together with a few of the other symptoms, and an examination revealed a decided prolapsus uteri, it was given in the 200th and followed by almost instant relief.

On the following morning I found her absolutely free from suffering and was told that she had had only one stool since the Lilium was given the evening before.

No other remedy was given afterward, and only two doses of the Lilium had been taken.

The following day she was discharged as cured.

I have long since learned to regard mental symptoms as of great importance in selecting remedies, and have often found them to be such "keynotes" as to overbalance some symptoms of apparently greater conspicuity. They often will suggest a remedy that has the other symptoms, which might not have been thought of but for the hint afforded by the mental condition.

